# High-beta oscillations at EEG resting state and hyperconnectivity of pain circuitry in fibromyalgia: an exploratory cross-sectional study

**DOI:** 10.3389/fnins.2023.1233979

**Published:** 2023-11-27

**Authors:** Rael Lopes Alves, Maxciel Zortea, Paul Vicuña Serrano, Rafaela Brugnera Tomedi, Rodrigo Pereira de Almeida, Iraci L. S. Torres, Felipe Fregni, Wolnei Caumo

**Affiliations:** ^1^Post-Graduate Program in Medical Sciences, School of Medicine, Universidade Federal do Rio Grande do Sul (UFRGS), Porto Alegre, Brazil; ^2^Laboratory of Pain and Neuromodulation, Hospital de Clínicas de Porto Alegre (HCPA), Porto Alegre, Brazil; ^3^Health School, University of Rio dos Sinos Valley (Unisinos), São Leopoldo, Brazil; ^4^Pharmacology of Pain and Neuromodulation: Pre-Clinical Investigations Research Group, Universidade Federal do Rio Grande do Sul (UFRGS), Porto Alegre, Brazil; ^5^Laboratory of Neuromodulation and Center for Clinical Research Learning, Physics and Rehabilitation Department, Spaulding Rehabilitation Hospital, Boston, MA, United States; ^6^Pain and Palliative Care Service, Hospital de Clínicas de Porto Alegre (HCPA), Porto Alegre, Brazil; ^7^Department of Surgery, School of Medicine, Universidade Federal do Rio Grande do Sul (UFRGS), Porto Alegre, Brazil

**Keywords:** fibromyalgia, pain connectome, EEG resting state, sLORETA, lagged coherence connectivity, BDNF

## Abstract

**Background:**

Electroencephalography (EEG) has identified neural activity in specific brain regions as a potential indicator of the neural signature of chronic pain. This study compared the lagged coherence connectivity between regions of interest (ROIs) associated with the pain connectome in women with fibromyalgia (FM) and healthy women (HC).

**Methods:**

We evaluated 64 participants (49 FM and 15 HC) during resting-state EEG sessions under both eyes open (EO) and eyes closed (EC) conditions. In addition to EEG measurements, we assessed clinical and psychological symptoms and serum levels of brain-derived neurotrophic factor (BDNF). The connectivity between eight ROIs was computed across eight different EEG frequencies.

**Results:**

The FM group demonstrated increased connectivity between the left dorsolateral prefrontal cortex (DLPFC) and right anterior cingulate cortex (ACC), specifically in the beta-3 frequency band (*t* = 3.441, *p* = 0.044). When comparing the EO and EC conditions, FM patients exhibited heightened interhemispheric connectivity between insular areas (*t*  = 3.372, *p* = 0.024) and between the left insula (INS) and right DLPFC (*t* = 3.695, *p* = 0.024) within the beta-3 frequency band. In the EC condition, there was a negative correlation between pain disability and connectivity in the beta-3 frequency band between the left ACC and the left primary somatosensory cortex (SI; *r* = −0.442, *p* = 0.043). In the EO condition, there was a negative correlation between central sensitization severity and lagged coherence connectivity in the alpha-2 frequency band between the right ACC and left SI (*r* = 0.428, *p* = 0.014). Moreover, in the EO–EC comparison, the lagged coherence connection between the left DLPFC and right INS, indexed by the gamma frequency band, showed a negative correlation with serum BDNF levels (*r* = −0.506, *p* = 0.012).

**Conclusion:**

These findings indicate that increased connectivity between different pain processing circuits, particularly in the beta-3 frequency band during rest, may serve as neural biomarkers for the chronic pain brain signature associated with neuroplasticity and the severity of FM symptoms.

## 1 Introduction

Fibromyalgia (FM) is a nociplastic pain syndrome characterized by widespread chronic musculoskeletal pain, fatigue, disrupted sleep, cognitive impairment, and mood disturbances (Wolfe et al., 2016). It ranks as the third most common musculoskeletal condition, increasing with age and being more common in women (Jones et al., 2015). FM is a primary chronic pain syndrome caused by either less inhibition of neurons in the medullary and supramedullary nociceptive pathways or more excitability and efficiency of synapses (Yunus, 2007).

The nociceptive system connects with multiple brain regions during pain perception modulated by interactions of ascending and descending pathways. These brain areas compose the pain network that includes the thalamic nuclei (TH), primary and secondary somatosensory cortices (SI and SII), insula (INS), anterior cingulate cortex (ACC), and prefrontal cortex (PFC; Apkarian et al., 2005). These structures collectively constitute a complex neural network, often called the pain matrix, involved in diverse processes such as motor withdrawal, attention, anticipation, memory, and habituation (Peyron and Fauchon, 2019).

The insula, situated deep within the lateral sulcus, between the frontal, temporal, and parietal lobes, acts as a convergence point for diverse information, serving as a hub where sensory, affective, and cognitive inputs converge. Specifically, the anterior insula regulates pain’s affective and motivational aspects, while the posterior insula is involved in processing sensory and discriminative aspects (Lu et al., 2016). The connection between the insula and the ACC acts as a salient stimulus switch, sending attention to the PFC, which connects to the default mode network (DMN) and the executive control network (CEN). Chronic pain patients may exhibit structural and functional connectivity changes within pain network regions (Kim and Kim, 2022).

The medial PFC also plays a role in pain control by sending direct projections to the periaqueductal gray (PAG), a key component of the descending pain modulatory system (DPMS; Kummer et al., 2020). Alterations in the PFC’s structure, activity, and connections have been associated with both acute and chronic pain (Ong et al., 2019). Functional near-infrared spectroscopy (fNIRS) measurements have indicated that increased activation of the left PFC following thermal stimulation is a sensitive indicator in FM patients with more severe clinical symptoms (Donadel et al., 2021).

The brain-derived neurotrophic factor (BDNF) plays a critical role in promoting the survival, growth, and plasticity of brain neurons. It is involved in various aspects of neural development, synaptic plasticity, and communication between neurons (Antal et al., 2010). The association between pathological conditions and serum BDNF levels, including depression, anxiety, and chronic musculoskeletal pain, has been established (Boulle et al., 2012; Caumo et al., 2017). Brain activity studies have explored the correlation effects with the BDNF. Theta and beta power positively correlate with serum BDNF levels in the right temporoparietal region in gambling disorder (Kim et al., 2022). Brain connectivity areas involved in pain processing, particularly functional connectivity between the thalamic subregions with the PFC, have been found to be associated with BDNF in individuals with long-term primary dysmenorrhea (Han et al., 2019). The Val/Met BDNF polymorphism has been linked to increased functional connections between the PFC and the motor cortex (MC) in FM patients. In contrast, the Val/Val polymorphism has been linked to reduced functional connections between the PFC and the MC, less active engagement of the DPMS, and FM symptoms that have a greater effect on the quality of life (de Oliveira Franco et al., 2022a). Therefore, it is important to observe possible correlations between serum BDNF levels and brain oscillation in pain-related areas in FM.

Electrophysiological data demonstrates that multiple pain-associated areas present overactivation in theta and low-beta brain oscillations in chronic neurogenic pain patients, which agrees with the concept of thalamocortical dysrhythmia (TCD) that predicts an increase in cortical brain oscillations related to thalamic deactivation (Stern et al., 2006; Ploner and May, 2018).

According to a recent review, electroencephalography (EEG) power spectral analysis of neuropathic pain studies has consistently demonstrated increased theta and high-beta bands and decreased high-alpha and low-beta bands, independent of pain intensity (Mussigmann et al., 2022). Moreover, patients with chronic neuropathic pain have exhibited diminished reactivity across broad EEG bands between eyes open (EO) and eyes closed (EC) conditions over the parieto-occipital region (Vuckovic et al., 2014). This relationship between EC and EO conditions concerning frequency bands and cortical locations provides a valuable physiological approach to analyzing brain functions (Mussigmann et al., 2022).

In another study, FM patients who used opioids on an as-needed basis showed reduced changes in peak amplitudes of EEG oscillations when transitioning from EO to EC conditions. This reduction was particularly pronounced in central theta, central beta, and parietal beta frequency bands. Reduced oscillatory activity in the parietal delta band of cortical activity was negatively correlated with pain-related disability, indicating that FM severity is associated with impaired cortical processing (Zortea et al., 2021). Analyzing brain electrical activity oscillations may serve as a sensitive and valuable approach to understanding the impact of neuroplasticity on disease severity.

Functional connectivity analysis using EEG data provides insights into how different brain regions communicate and work together. One method commonly used for source localization and functional connectivity analysis of EEG data is standardized low-resolution electromagnetic tomography (sLORETA). sLORETA is a distributed source localization method that estimates the neural sources of EEG signals by solving the inverse problem. It uses a three-dimensional head model and EEG recordings to estimate electrical activity in different brain regions. This method was validated through correlations with other imaging techniques, such as functional magnetic resonance imaging (fMRI) and MRI (Mulert et al., 2004; Kim et al., 2022). Despite the use of a limited number of electrodes, sLORETA has provided valuable insights into the underlying sources of brain activity (Michel and Brunet, 2019). In recent years, several studies have supported the use of sLORETA to explore cortical activity patterns and functional connectivity in various psychiatric and neurological conditions, including bipolar disorder, autism spectrum disorder (ASD), obsessive-compulsive disorder (OCD), tinnitus, and FM (Vanneste et al., 2011; Coben et al., 2014; Vanneste et al., 2017; Yoshimura et al., 2018; Painold et al., 2020).

Functional EEG connectivity has been employed to investigate the interaction among brain structures implicated in chronic pain. The functional dynamics of brain networks in FM patients demonstrate inhibition of the connectivity between the DLPFC, DMN, and descending pain pathways. These connectivity effects were obtained mainly in the alpha-2 frequency band, which indicates the integration of pain in the self-perception characterizing the chronic pain condition (Vanneste et al., 2017).

In a study examining pain in sickle cell disease (SCD) using the sLORETA approach, patients exhibited decreased theta activity in the precuneus while showing increased theta and beta-2 activity in areas related to pain processing, such as the PFC, ACC, the left operculum-insular region and caudate nucleus. The increased theta activity observed in SCD patients is likely caused by the TCD mechanism (Case et al., 2018). Another study assessing patients with radicular or musculoskeletal chronic pain revealed heightened activation of theta and low-alpha bands across various brain regions in the left hemisphere (Prichep et al., 2018).

Functional connectivity describes the pattern of interaction in terms of the statistical methods, correlation, or covariance between different anatomical locations. The coherence method calculates the slope of the phase difference spectrum (phase-lag), providing estimates of the time delay between corresponding time series across a range of frequencies (Chiarion et al., 2023). Regarding EEG coherence source analysis, the sLORETA approach may offer a more comprehensive and accurate assessment than other pairwise measurement techniques (Coben et al., 2014).

Based on the information provided, the objective of this study was to investigate whether the connectivity of regions of interest (ROIs) involved in pain processing differs between FM patients and healthy controls (HC). The study also examined the relationship between brain oscillations in these ROIs and clinical, psychological, and serum BDNF levels in FM patients. By analyzing these relationships, the researchers sought to gain insights into the neural correlates of FM and potentially identify neural markers associated with the severity of clinical symptoms.

## 2 Methods

### 2.1 Study design and settings

We conducted a cross-sectional study and used the Strengthening the Reporting of Observational Studies in Epidemiology (STROBE) guidelines to report the methods and results, which were approved by the Research Ethics Committee at the Hospital de Clínicas de Porto Alegre (HCPA) under the registration number (2020-0369) according to the international ethical standards based on the Declaration of Helsinki. The study was conducted in accordance with the relevant guidelines and regulations. All participants provided written informed consent before participating in this study.

The study started in September 2019 and stopped due to the COVID-19 pandemic in March 2020. The study restarted in November 2020 with several modifications and restrictions to prioritize the safety of participants and researchers. Data collection was completed in November 2022.

### 2.2 Participants, recruitment, inclusion, and exclusion criteria

Participants were recruited from the outpatient pain clinic of the HCPA Pain Service, the Basic Health Unit, and across the media. They were women aged 30–65 years diagnosed with FM, according to the American College of Rheumatology (ACR) 2016 (Wolfe et al., 2016). They needed to be literate and report a score of 6 or higher on the Numerical Pain Scale (NPS 0–10) most of the time in the last 3 months. A team of physicians with extensive experience in pain management confirmed the diagnosis. Participants were excluded from the study if they had used alcohol or drugs in the last 6 months, were pregnant, had a neurological disease, or had a history of head trauma or neurosurgery. Additional exclusion criteria were if the patients had decompensated systemic diseases, chronic inflammatory diseases, uncompensated hypothyroidism, another metabolic disease, or were receiving cancer treatment.

In this study, screening was performed on 133 FM participants who were eligible to participate. However, 67 did not meet the inclusion criteria for different reasons, such as living far away from the research center, having trouble getting around on public transportation, and being unemployed. Some screened participants did not meet the diagnostic criteria for FM. In addition, they were excluded if they met the following diagnosis criteria: their pain levels were lower than 6 (NPS 0–10) or if they had another uncompensated clinical disease (rheumatoid arthritis, lupus, hypothyroidism, etc.). Thus, 66 FM were included in the study, but 17 were excluded because of low-quality EEG signals. Thus, in the end, 49 subjects were included in the analysis.

The HC subjects were literate women aged 30–65 years. They were recruited from the local community through social media. They had to take a phone test to ensure they were not sick or on medicine. In the HC, 33 participants were screened, and 16 were included. Seventeen people were excluded from the study because their Beck Depression Inventory-II (BDI-II) score was higher than 13 (Gomes-Oliveira et al., 2012) or because they regularly took painkillers, antidepressants, anticonvulsants, anxiety-reducers, hypnotics, etc. In addition, one subject was excluded from the EEG preprocessing data because of low-quality signals. Thus, the final sample of HC comprised 15 participants.

The final sample comprised 64 participants (49 FM and 15 HC). Demographic and clinical measures are presented in Table 1.

**Table 1:** Demographic and clinical characteristics of the study sample.

	FM group (*N* = 49)		HC group (*N* = 15)		
	Mean (SD) or RF (%)	Median (IQ 25–75)	Mean (SD) or RF (%)	Median (IQ 25–75)	*p-*values
**Demographic measures**					
Age (years)	48.06 (9.83)	47.00 (40.50, 56.00)	41.47 (8.42)	40.00 (34.00,50.00)	<0.05
Years of formal study	12.07 (4.00)	11.00 (9.25, 15.50)	15.73 (5.06)	17.00 (11.00, 20.00)	<0.05
American College of Rheumatology (ACR) diagnosis tool	22.78 (3.74)	23 (19.50, 25.00)	-	-	-
Employed (yes/no)%	29/20 (59.2%)	-	12/3 (80.0%)	-	0.220
Smoking^*^ (yes/no)%	12/37 (24.5%)	-	0/15 (0.0%)	-	0.054
Drinking^*^ (yes/no)%	24/25 (49.0%)	-	10/5 (66.7%)	-	0.225
**Clinical comorbidity**					
HAS (yes/no)	15/34 (30.0%)	-	3/12 (20.0%)	-	-
Cardiac disease (yes/no)	2/47 (4.1%)	-	1/14 (6.7%)	-	-
Diabetes disease (yes/no)	4/45 (8.2%)	-	0/15 (0.0%)	-	-
Hypothyroidism (yes/no)	7/42 (14.3%)	-	0/15 (0.0%)	-	-
Asthma (yes/no)	12/37 (24.5%)	-	0/15 (0.0%)	-	-
Epilepsy (yes/no)	0/49 (0.0%)	-	0/15 (0.0%)	-	-
Renal insufficiency (yes/no)	1/48 (2.0%)	-	0/15 (0.0%)	-	-
**Biochemical measures**					
Serum BDNF (pg/mL)	45.25 (32.87)	31.64 (21.16, 68.65)	-	-	-
**Mood, pain, and sleep quality measures**					
Beck Depression Inventory (BDI-II)	24.82 (11.01)	24.00 (14.50, 34.50)	-	-	-
Brazilian Portuguese Central Sensitization Inventory (BP-CSI)	62.90 (13.88)	65.00 (52.00, 75.50)	-	-	-
Brazilian Portuguese Pain Catastrophizing Scale	35.57 (10.88)	36.00 (29.00, 44.00)	-	-	-
Pittsburgh Sleep Quality Index (total score)	12.22 (3.53)	13.00 (9.50, 14.00)	-	-	-
Fibromyalgia Impact Questionnaire (FIQ; total score)	67.41 (16.66)	71.57 (62.17, 78.40)	-	-	-
**Psychiatric disorder**					
Major Depressive Disorder (MDD; current; yes/no)	24/25 (49.0%)	-	-	-	-
Generalized Anxiety Disorder^a^ (GAD; yes/no)	10/39 (20.4%)	-	-	-	-
**Medication use**					
Antidepressant (yes/no)	29/20 (59.2%)	-	-	-	-
Anticonvulsant (yes/no)	11/38 (22.4%)	-	-	-	-
Benzodiazepines (yes/no)	7/42 (14.3%)	-	-	-	-
Opioid analgesic (yes/no)	13/36 (26.5%)	-	-	-	-
Non-opioid analgesic (yes/no)	45/4 (91.8%)	-	-	-	-
HAS^a^ (yes/no)	12/37 (24.5%)	-	-	-	-

Data are presented as mean and standard deviation (SD) and as median and interquartile (*n* = 64) or relative frequencies (RF) and percentage (*n* = 64).

### 2.3 Instruments and assessment of outcomes

#### 2.3.1 Dependent and independent variables

**Figure 1:**
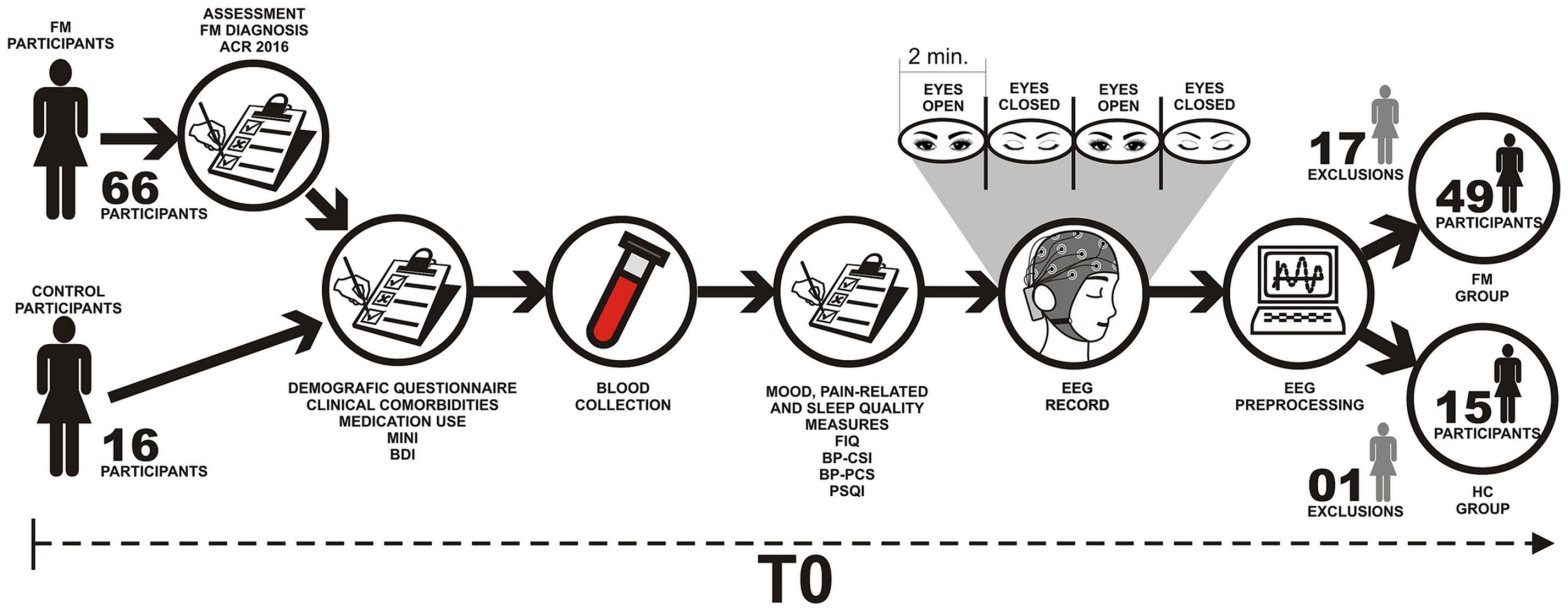
Flowchart of the study assessments. FM, Fibromyalgia; HC, Health Controls; ACR, American College of Rheumatology; FIQ, Fibromyalgia Impact Questionnaire; BDI, Beck Depression Inventory; MINI, Mini International Neuropsychiatric Interview; BP-PCS, Pain Catastrophizing Scale; BP-CSI, Central sensitization inventory—Brazilian Portuguese version; PSQI, Pittsburgh Sleep Quality Index.

The dependent variables (outcome) were the lagged coherence between ROIs of the pain connectome assessed by delta, theta, alpha-1, alpha-2, beta-1, beta-2, beta-3, and gamma EEG frequencies in the resting state. Other variables of interest were evaluated in FM subjects, such as pain intensity, BDNF serum levels, pain catastrophizing, depressive symptoms, sleep quality, demographic characteristics, clinical and psychiatric chronic diseases, and psychotropic and analgesic medications. The sequence of assessments is presented in Figure 1.

##### 2.3.1.1 Assessment of the primary outcome

- *EEG recording:* Assessments were conducted in a quiet room with the subjects sitting in a comfortable armchair. EEG was recorded using 18 scalp sites according to the 10–20 system (Jurcak et al., 2007). FP1, FP2, F7, F3, Fz, F4, F8, T7, C3, Cz, C4, T8, P7, P3, Pz, P4, P8, Oz, and the left ear (EXT), with reference to the right ear (CMS/DRL). The EEG system was the ENOBIO 20, made by Neuroelectrics in Barcelona, Spain. It has a cap with 1.75 cm^2^ gel electrodes in a circle. The impedance was 5 kΩ for all electrodes, with a high dynamic resolution (24 bits, 0.05 uV) and a sampling rate of 500 Hz. A line noise filter (60-Hz) was applied to remove the main line artifacts from the EEG data.
- *Resting-state paradigm:* Resting-state EEG was collected for 8 min, with 2 min switched between EC and EO conditions. Participants were instructed to remain awake, relaxed, and thinking-free. During the EO condition, the participants were instructed to keep their eyes on a black cross fixed on the front wall at eye level 1.5 m ahead of the armchair. The EC condition is the level of arousal at rest, and the EO condition is the level of arousal at activation. The difference between EO and EC represents the activation process (Barry et al., 2007).

##### 2.3.1.2 Preprocessing and functional connectivity analysis

The EEG data were cleaned using the open-source toolbox EEGLAB 14.1 (Delorme and Makeig, 2004), which ran in the MATLAB environment (The MathWorks Inc., Natick, Massachusetts, United States). Visual inspection was performed for artifact detection, and segments of bad channels were removed if necessary. Continuous EEG data were band-pass filtered using a simple FIR filter with cutoff frequencies of 0.5–40 Hz, resampled to 250 Hz, and split into 4.096 s epochs (Ponomarev et al., 2014).

Rejection thresholds were determined according to the artifact attributes. To eliminate eye blinks and other quick movements from nonfiltered continuous EEG, 50 μV thresholds were set for the FP1 and FP2 electrodes and 100 μV thresholds for the other electrodes. To eliminate artifacts associated with slow head or body movements, 50 μV thresholds were used for slow waves (0–1 Hz band), while 30 μV thresholds were used for fast waves (20–35 Hz band). Epochs containing artifacts were automatically excluded from the analysis (Jäncke and Alahmadi, 2016). The mean size of the data after artifact rejections according to conditions was 107 s for the HC group in the EO condition, 140 s for the HC group in the EC condition, 123 s for the FM group in the EO condition, and 146 s for the FM group in the EC condition. To compute brain connectivity, the minimum threshold was fixed at 40 s for each resting-state condition (EO, EC, and the difference between EO and EC; Ponomarev et al., 2014). Subjects or conditions below this threshold were excluded from the analysis.

Functional connectivity measures were estimated using the sLORETA algorithms, which compute the linear dependence (coherence) of electric neuronal activity from several brain regions (Pascual-Marqui et al., 1994; Pascual-Marqui, 2002; Pascual-Marqui, 2007a). Lagged coherence connectivity expresses the coherence measured by the corrected standardized covariance of scalp electric potentials, extracting instantaneous, non-physiological effects due to volume conduction and the low spatial resolution of EEG (Pascual-Marqui, 2007a; Pascual-Marqui, 2007b).

The sLORETA functional connectivity images of lagged coherence were computed in the following discrete frequency bands identified via factor analysis (Kubicki et al., 1979; Paszkiel, 2020): delta (1–3.5 Hz), theta (4–7.5 Hz), alpha-1 (8–10 Hz), alpha-2 (10–12 Hz), beta-1 (13–18 Hz), beta-2 (18.5–21 Hz), beta-3 (21.5–30 Hz), and gamma (30.5–44 Hz).

The EEG electrode coordinates employed by the software are based on the MRI anatomical template from the Montreal Neurological Institute (MNI152), which slices and classifies the neocortical volume (limited to the gray matter) in 6,239 voxels of dimension 5 mm^3^ (Mandal et al., 2012).

A voxel-wise approach was used to identify the ROIs, and the MNI coordinates of the areas beneath the electrode were determined using sLORETA. The ROIs were established as 10-mm-diameter spheres (Coben et al., 2014; Bosch-Bayard et al., 2022), centered on the peak coordinates obtained from the seed points. These seed points included the left and right primary somatosensory cortex (BA01; Dai et al., 2018), the left (Wang et al., 2022) and right (Cifre et al., 2012) insular cortex (BA47-BA48), the left and right anterior cingulate cortex (BA24; Fauchon et al., 2020), and the left and right dorsolateral prefrontal cortex (BA09-BA10-BA46; Fauchon et al., 2020), as indicated by previous studies on the pain network. The specific coordinates are presented in Table 2.

**Table 2:** Regions of interest (ROIs).

Seed	MNI coordinates			Brodmann area
	x	y	z	
Left SI	−21	−35	68	BA01 (trunk region)
Right SI	20	−33	69	
Left INS	−36	16	−6	BA47 and BA48 (anterior region)
Right INS	36	6	6	
Left ACC	−3	36	16	BA24
Right ACC	2	36	16	
Left DLPFC	−35	40	−24	BA09, BA10 and BA46
Right DLPFC	34	42	24	

ACC, anterior cingulate cortex; INS, insula; DLPFC, dorsolateral prefrontal cortex; SI, primary somatosensory cortex.

We employed the following steps to characterize changes in EEG-lagged coherence connectivity across ROIs:

*Step 1*: Independent group comparison of lagged coherence—We compared the lagged coherence between the FM and HC groups in two arousal states, EC and EO. The independent group test was performed to assess whether FM_(EC)_ = HC_(EC)_ and FM_(EO)_ = HC_(EO)_.
*Step 2:* Independent group comparison of lagged coherence—We examined the difference in lagged coherence between the EC and EO conditions (EO–EC) within the FM and HC groups. The independent group test was conducted to determine if (FM_(EO)_ − FM_(EC)_) = (HC_(EO)_ − HC_(EC)_).
*Step 3:* Regression analysis of lagged coherence with independent variables (IV)—In the FM group, we performed regression analysis to explore the relationship between EEG-lagged coherence connectivity and IV in both EC and EO conditions. Single regression analyses were conducted for FM_(EC)_ vs. IV, FM_(EO)_ vs. IV, and the difference between the EC and EO conditions (EO–EC). Paired contrasts were performed for FM_(EO–EC)_ vs. IV.

#### 2.3.2 Assessment of clinical, psychological, and biochemical variables

The tools used to measure psychological and clinical measures were validated in the Brazilian population, and the assessment was performed by psychiatrists and trained psychologists.

- The sociodemographic questionnaire contains information related to age, years of study, clinical diagnoses, health problems (self-reported), and medication use.
- Mini-International Neuropsychiatric Interview (MINI) is a short (15–30 min) structured diagnostic interview aimed at screening for DSM-IV and ICD-10 diagnoses. In the present study, we reported information related to major depressive and manic episodes, panic disorder, social phobia, OCD, post-traumatic stress disorder, and generalized anxiety disorder (Amorim, 2000).
- The Beck Depression Inventory (BDI) is a self-report questionnaire that evaluates depressive symptom severity (Gomes-Oliveira et al., 2012).
- The Brazilian Portuguese translation of the Pain Catastrophizing Scale (BP-PCS) was used to assess the emotional dimension of pain and measure how patients perceive it. It is divided into three domains: magnification, helplessness, and rumination, and questions are asked to determine the patient’s feelings and thoughts when they are in pain (Sehn et al., 2012).
- The central sensitization inventory (CSI) is a tool that identifies key symptoms related to central sensitization processes by quantifying their severity. Part A is a 25-item self-report questionnaire designed to assess health-related symptoms, and Part B (not rated) is designed to determine the presence of one or more specific disorders (Caumo et al., 2017).
- The Fibromyalgia Impact Questionnaire (FIQ) was used to assess how the quality of life is negatively influenced by FM clinical conditions. The questionnaire is composed of 10 items with scores of 0 to 10. Therefore, the maximum score is 100. Higher scores denote a greater impact of FM symptoms on quality of life (Marques et al., 2006).
- The Pittsburgh Sleep Quality Inventory (PSQI) measures sleep quality. This self-reported instrument was used to assess the sleep quality and sleep disturbances that were present over a month through questions about how long it takes to fall asleep, how long people sleep, how they feel about the quality of their sleep, if they take sleeping pills, if they have trouble sleeping during the day, how well they sleep, and if they have problems sleeping. The sum of these items classified the subjects into two groups: good sleepers and poor sleepers (Bertolazi et al., 2011).
- Enzyme-linked immunosorbent assay (ELISA) monoclonal antibodies specific for BDNF were used to measure the blood levels of BDNF (R&D Systems, MN, United States; ChemiKine BDNF Sandwich ELISA kit, CYT306; Chemicon/Millipore, Billerica, MA, United States). The inter-assay variance was performed using two plates per kit on 2 days during the same week. All procedures adhered to the manufacturer’s recommendations, with 7.8 pg./mL being the lowest detection limit for BDNF. ELISA was performed at an optical density of 450 nm (Promega, WI, USA; GloMax®-Multi Microplate Reader). Multiplexing assay measurements were performed using a Bio-Plex®-200 instrument (Bio-Rad). Total protein was assessed using bovine serum albumin following the Bradford method.

### 2.4 Sample size estimation

The sample estimation was based on a prior study that evaluated speech decoding using ANOVA (2 groups × 3 conditions × 4 blocks). Functional connectivity (lagged coherence) was the dependent variable (outcome) measured by EEG between bilateral auditory-related cortical areas (ARCAs) and the Broca’s area (Elmer et al., 2017). This study found an Eta Square = 0.15 (Cohen’s *d* = 0.84) to an alpha of 0.05 and a power of 0.80. For an independent *t*-test, we determined a sample size of 72 participants according to an allocation ratio of 4:1 between FM and HC. We increased the sample size by 15%, bringing the total to 82 cases (66 FM and 16 HC). This was done to guarantee the power of the study due to possible unexpected events. During the EEG preprocessing data, 17 FM and one HC were excluded because of low-quality EEG signals. Thus, 49 FM subjects and 15 HC were included in the analysis. Sample size estimation was performed using G*Power 3 software (Faul et al., 2007).

Given that this study is exploratory in nature and that the sample size was estimated *a priori*, the power of the analysis was reassessed to ensure the robustness of the results. Based on this assumption, and according to the mean (standard deviation) of lagged coherence connectivity between the left DLPFC and right ACC in the FM group 20.60 (2.22) compared with the HC group 18.19 (2.87), the effect size was *d* = 0.93 for an alpha error lower than 5% and power of 0.87 (means and SD values multiplied by 10^−4^).

### 2.5 Statistical analysis

Descriptive statistics were utilized to provide a summary of the primary demographic characteristics of the sample. The normality of data distribution was assessed using the Shapiro–Wilk test. To compare continuous variables between groups, independent sample *t*-tests were employed. Categorical variables were compared between groups using the chi-square test and Fisher’s exact tests.

Considering the imbalance on age and education between the FM and HC groups, we performed a linear regression model using the stepwise forward method between groups, adjusting the average of ROIs as a dependent variable in all frequency bands for years of formal education and age as covariates. We transpose each matrix of each participant and perform multiple regression for each ROI in all frequency bands for both conditions, EO and EC. A *value of p* of 0.05 was required to include the covariates in the model. We used the adjusted value for the ROIs whose difference was significant to rebuild all matrices and transpose them to the Loreta package for lagged coherence connectivity analysis between groups. Multiple regression analysis was performed using SPSS software version 22.0 (SPSS, Chicago, IL, United States).

For the independent group analysis of lagged coherence, we employed the sLORETA package, which utilizes nonparametric statistical analyses to compute functional connectivity nodes in lagged coherence for each frequency band across the eight ROIs. To establish contrasts, we performed statistical nonparametric mapping (SnPM) using a *t*-statistic for unpaired groups, with corrections for multiple comparisons. The significance threshold was determined based on a randomization test involving 5,000 permutations. This nonparametric approach is an alternative method rooted in permutation test theory, eliminating the need for Gaussian assumptions while correcting for multiple comparisons (Nichols and Holmes, 2002).

Regression analysis of the lagged coherence was exclusively conducted on FM patients, focusing on lagged coherence for each frequency band between ROIs (the dependent variable) and various independent variables, including FIQ, CSI, BDI, BP-PCS, PSQI, and serum BDNF levels. Linear regressions were computed separately for the EO, EC, and EO–EC conditions. SnPM with 5,000 permutations was employed to determine the significance threshold and correct for multiple comparisons.

## 3 Results

The demographic measures present significant differences in years of formal study and age between the FM and HC groups, as demonstrated in Table 1.

### 3.1 Evaluation of lagged coherence connectivity in FM and HC according to EO and EC conditions

Tables 3, 4 show regression analysis using linear regression analyses following the stepwise method to adjust for years of education level and age according to according to the FM and HC groups. We adjusted each ROI in the EO condition for years of education level and age using linear regression analyses following the stepwise method. The variables of age and education level were retained in the regression models only when they correlated with ROIs that showed a statistically significant difference (*p* < 0.05). We found that age and education level were negatively correlated in the delta and beta-3 frequency bands, whereas they were positively correlated in the alpha-1 and gamma frequency bands, mainly involving the connectivity between the insula with ACC and DLPFC. In the EC condition, age and education levels were negatively correlated with ROIs in the delta, beta-1, and beta-2 frequency bands and positively correlated between ROIs in the alpha-1, beta-3, and gamma bands involving connections between the insula and SI with ACC and DLPFC.

**Table 3:** Linear regression model by stepwise forward method for each ROIs adjusted for years of formal study and age between the FM and HC groups in the EO condition (*n* = 64).

EO CONDITION								
	β	Std error	β^Sd^	t	*p*	CI 95%	Predicted mean^*^ (SD^*^)	Adjusted mean^*^ (SD^*^)
**Frequency band (delta)**								
Dependent variable: ROI (INS left ↔ ACC left)							308,785 (4,445)	308,749 (4,472)
Age	−450.34	200.4	−0.274	−2.247	0.028	−851.06 to-49.62		
Dependent variable: ROI (INS right ↔ DLPFC left)							320,514 (5,398)	320,472 (5,431)
Age	−546.93	258.1	−0.260	−2.119	0.038	−1062.9 to-30.90		
Dependent variable: ROI (INS right ↔ ACC left)							273,976 (4,844)	273,927 (4,885)
Age	−490.7	229.9	−0.262	−2.134	0.037	−950.3 to-31.14		
**Frequency band (Alpha-1)**								
Dependent variable: ROI (SI left ↔ INS right)							3574.6 (436.2)	3572.3 (442.9)
Formal education	96.74	42.48	0.278	2.277	0.026	11.82 to 181.6		
**Frequency band (beta-3)**								
Dependent variable: ROI (INS right ↔ ACC right)							1260.8 (54.35)	1261.1 (55.50)
Formal education	−12.05	5.87	−0.252	−2.2054	0.044	−23.78 to −0.322		
**Frequency band (gamma)**								
Dependent variable: ROI (SI left ↔ DLPFC right)							4072.7 (122.9)	4073.1 (124.8)
constant	3207.4	287.65		11.150	0.000	2,632 to 3,782		
Age	12.94	4,47	0.378	2.890	0.005	3.98 to 21.90		
Formal education	20.35	9.80	0.272	2.076	0.042	0.75 to 39.96		
Dependent variable: ROI (SI right↔ DLPFC right)							4420.0 (89.75)	4420.1 (90.57)
Age	9.09	4.40	0.254	2.065	0.043	0.28 to 17.89		
Dependent variable: ROI (INS left ↔ DLPFC right)							934.78 (35.40)	934.42 (35.76)
Age	3.58	1.587	0.276	2.260	0.027	0.41 to 6.75		
Dependent variable: ROI (INS right ↔ DLPFC left)							2477.5 (79.26)	2477.5 (81.04)
Formal education	17.57	7.96	0.270	2.206	0.031	1.65 to 33.50		
Dependent variable: ROI (INS right ↔ ACC left)							934.78 (35.40)	934.42 (35.76)
Formal education	17.44	6.55	0.320	2.662	0.010	4.32 to 30.54		
Dependent variable: ROI (INS right ↔ ACC right)							2119.8 (46.03)	2119.6 (46.67)
Formal education	10.20	4.69	0.266	2.176	0.033	0.83 to 19.58		

β, Unstandardized Coefficients; β^sd^, Beta Standardized. ACC, anterior cingulate cortex; INS, insula; DLPFC, dorsolateral prefrontal cortex; SI, primary somatosensory cortex. ^*^Values multiplied for (10^6^). ^**^p < 0.05.

**Table 4:** Linear regression model by stepwise forward method for each ROIs adjusted for years of formal study and age between the FM and HC groups in the EC condition (*n* = 64).

EC CONDITION								
	β	Std error	β^Sd^	T	*p*	CI 95%	Predicted mean^*^ (SD^*^)	Adjusted mean^*^ (SD^*^)
**Frequency band (delta)**								
Dependent variable: ROI (SI left ↔ INS left)							134,853 (5,329)	134,908 (5,349)
Age	−539.93	204.11	−0.318	−2.645	0.010	−947.9 to −131.9		
Dependent variable: ROI (SI left ↔ ACC right)							212,996 (2,551)	213,026 (2,563)
Age	−258.47	111.44	−0.283	−2.319	0.024	−481.2 to −35.7		
Dependent variable: ROI (SI right ↔ INS left)							263,788 (9,221)	263,929 (9,250)
Age	−934.26	383.36	−0.266	−2.437	0.018	−1,700 to −167		
Dependent variable: ROI (INS left ↔ DLPFC left)							254,675 (7,310)	254,802 (7,369)
Age	−740.60	352.59	−0.257	−2.095	0.040	−1,447 to −33.78		
Dependent variable: ROI (INS left ↔ DLPFC right)							249,394 (8052)	249,513 (8070)
Age	−815.79	308.38	−0.318	−2.645	0.010	−1,432 to −199.3		
Dependent variable: ROI (INS left ↔ ACC left)							306,061 (7,214)	306,167 (7,227)
Age	−730.88	310.59	−0.286	−2.353	0.022	−1,351 to −110.0		
Dependent variable: ROI (INS left ↔ ACC right)							349,631 (8,654)	349,777 (8,689)
Age	−876.82	384.04	−0.278	−2.283	0.026	−1,644 to −109		
Dependent variable: ROI (ACC left ↔ ACC right)							465,832 (7,000)	465,933 (7,055)
Age	−70.21	347.10	−0.251	−2.043	0.045	−1,403 to −15.36		
**Frequency band (alpha-1)**								
Dependent variable: ROI (SI right ↔ INS right)							1463.34 (69.84)	1461.91 (69.64)
Age	7.077	3.519	0.247	2.011	0.049	0.042 to 14.11		
Dependent variable: ROI (INS left ↔ INS right)							1378.44 (138.40)	1378.92 (136.73)
Formal education	30.694	13.367	0.280	2.296	0.025	3.97 to 57.41		
Dependent variable: ROI (INS left ↔ ACC right)							7983.54 (177.36)	7983.22 (179.04)
Formal education	39.336	19.503	0.248	2.017	0.048	0.350 to 78.32		
**Frequency band (alpha-2)**								
Dependent variable: ROI (SI left ↔ INS left)							597,403 (15,888)	16,030 (16,030)
Age	−1609.6	793.93	−0.249	−2.027	0.047	−3,196 to −22.60		
Dependent variable: ROI (INS left ↔ INS right)							223,576 (4,305)	223,662 (43,41)
Age	−436.18	212.37	−0.252	−2.054	0.044	−860.7 to −11.66		
**Frequency band (beta-1)**								
Dependent variable: ROI (SI left ↔ ACC right)							48995.6 (455.9)	49002.2 (454.1)
Age	−46.192	18.465	−0.303	−2.502	0.015	−83.10 to −9.28		
Dependent variable: ROI (INS left ↔ DLPFC left)							63278.0 (789.8)	63287.4 (787.7)
Age	−80.016	32.804	−0.296	−2.439	0.018	−145.59 to −14.44		
**Frequency band (beta-2)**								
Dependent variable: ROI (SI left ↔ ACC right)							221,818 (4,050)	221,879 (4,103)
Age	−410.39	196.42	−0.256	−2.099	0.041	−803.0 to −17.74		
Dependent variable: ROI (SI right ↔ ACC right)							319,276 (5,927)	319,341 (5,959)
Age	−600.49	252.40	−0.288	−2.370	0.021	−1,107 to −93.94		
**Frequency band (beta-3)**								
Dependent variable: ROI (SI right ↔ DLPFD left)							868.89 (118.59)	868.55 (119.44)
Age	12.015	5.707	0.258	2.105	0.039	0.607 to 23.42		
Dependent variable: ROI (SI right ↔ DLPFC right)							598.36 (52.29)	598.54 (52.70)
Age	5.298	2.220	−0.290	2.386	0.020	0.86 to 9.73		
Dependent variable: ROI (INS left ↔ INS right)							706.30 (23.47)	706.37 (23.42)
Formal education	5.205	2.087	0.302	2.495	0.015	1.03 to 9.37		
Dependent variable: ROI (INS left ↔ DLPFC right)							2414.2 (112.58)	2413.9 (112.92)
Formal education	24.969	10.920	0.279	2.287	0.026	3.14 to 46.79		
Dependent variable: ROI (ACC left ↔ ACC right)							796.74 (44.49)	796.67 (44.91)
Age	4.507	2.138	0.259	2.108	0.039	0.234 to 8.781		
**Frequency band (gamma)**								
Dependent variable: ROI (SI left ↔ DLPFD left)							3556.6 (211.92)	3554.82 (212.96)
Age	21.470	9.589	0.274	2.239	0.029	2.302 to 40.63		
Dependent variable: ROI (SI left ↔ DLPFD right)							3896.4 (124.0)	3895.5 (124.7)
Age	12.566	6.181	0.250	2.033	0.046	0.209 to 24.92		
Dependent variable: ROI (SI left ↔ ACC right)							1653.6 (40.29)	1653.2 (40.12)
Formal education	−8.936	3.845	−0.283	−2.324	0.023	−16.62 to −1.25		
Dependent variable: ROI (SI right ↔ DLPFC left)							3669.7 (212.8)	3667.9 (213.9)
Age	21.563	9.460	0.278	2.279	0.026	2.65 to 40.47		
Dependent variable: ROI (SI right ↔ ACC left)							1390.0 (54.83)	1389.9 (54.42)
Formal education	−12.16	5.163	−0.287	−2.356	0.022	−22.48 to −1.84		
Dependent variable: ROI (INS right ↔ ACC left)							2178.2 (71.64)	2177.8 (71.69)
Formal education	−15.888	7.640	−0.255	−2.080	0.042	−31.15 to-0.61		
Dependent variable: ROI (DLPFC left ↔ ACC left)							2163.5 (159.2)	2163.0 (160.0)
Age	16.137	7.190	0.274	2.245	0.028	1.76 to 30.50		
Dependent variable: ROI (DLPFC left ↔ ACC right)							2121.8 (81.06)	2121.6 (81.75)
Age	8.213	3.956	0.255	2.076	0.042	0.30 to 16.12		

β, Unstandardized coefficients; β^sd^, Beta Standardized. ACC, anterior cingulate cortex; INS, insula; DLPFC, dorsolateral prefrontal cortex; SI, primary somatosensory cortex. ^*^Values multiplied for (10^6^). ^**^p < 0.05.

### 3.2 Analysis of lagged coherence connectivity in the EO, EC conditions according to FM and HC

**Figure 2:**
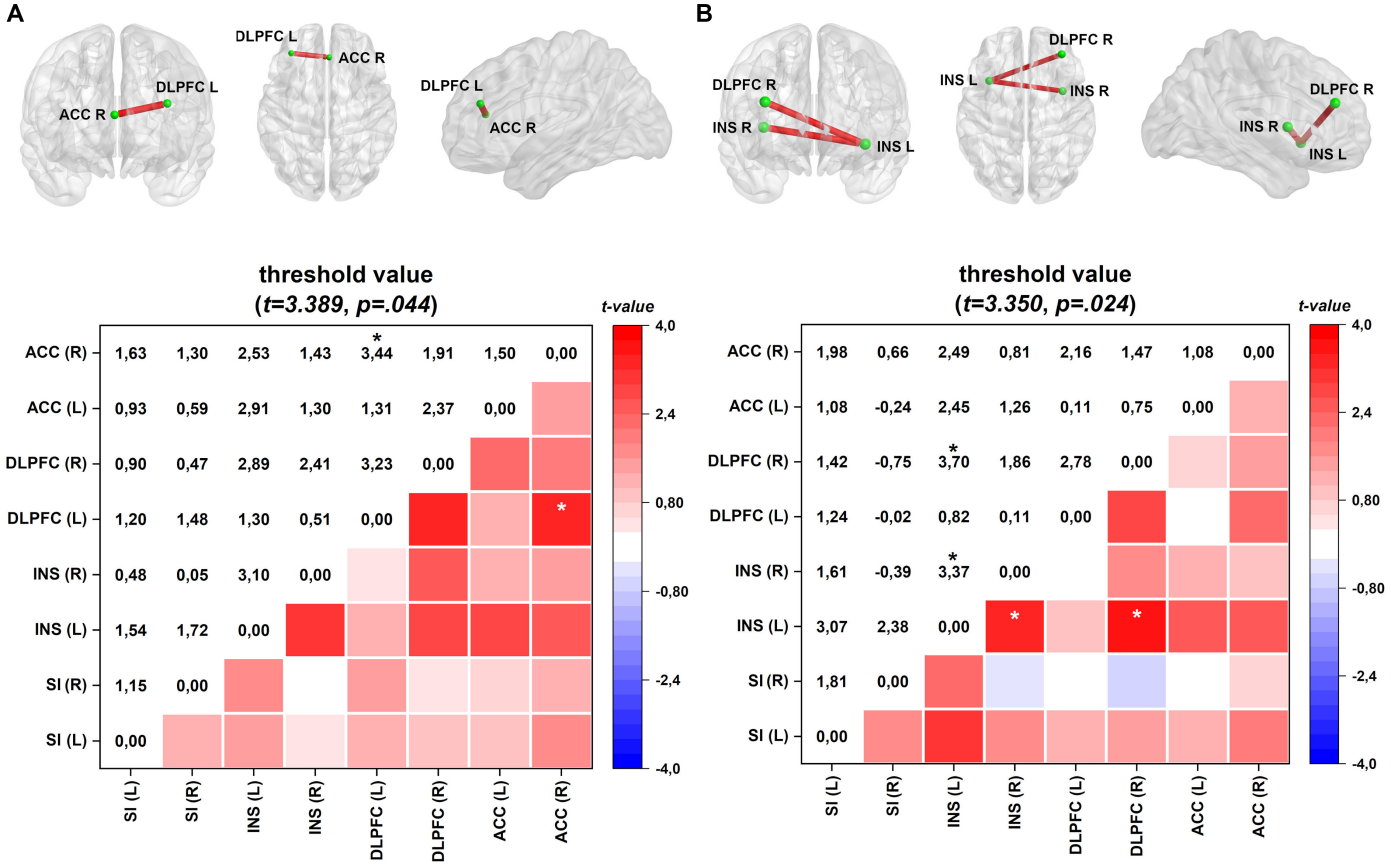
Connectivity (frontal, axial, and sagittal planes) and color maps of the FM group compared with those of the HC group. **(A)** In the EO condition, the FM group exhibited increased lagged coherence connectivity between the right ACC and left DLPFC in the beta-3 frequency band (Cohen’s d = 0.44). **(B)** In the activation process, difference between EO and EC conditions, FM group shows enhanced connectivity between left and right insula and between the left insula and right DLPFC in the beta-3 frequency band (Cohen’s d = 0.44). Effect sizes are based on Cohen’s d: small = 0.2, medium = 0.5, large = 0.8. The colored edge represents connections with significant differences. Values are given as t-values. ACC, anterior cingulate cortex; INS, insula; DLPFC, dorsolateral prefrontal cortex; SI, primary somatosensory. ^*^p < 0.05.

Figure 2 depicts the lagged coherence connectivity in the EO, EC, and the difference between the EO and EC conditions for comparisons between FM and HC subjects. In the EC condition, there were no significant differences in lagged coherence connectivity between the FM and HC groups.

However, during the EO condition in FM, there was a notable increase in lagged coherence connectivity in the beta-3 frequency band between the left dorsolateral prefrontal cortex (DLPFC) and the right ACC. This enhanced connectivity is presented in Figure 2A.

Furthermore, when comparing the difference between EO and EC conditions (EO–EC), FM patients exhibited heightened interhemispheric connectivity in the beta-3 band between the left and right insular areas and between the left insula and right DLPFC. Increased connectivity is shown in Figure 2B.

### 3.3 Relationship between lagged coherence connectivity and severity of clinical symptoms and BDNF in FM subjects

**Figure 3:**
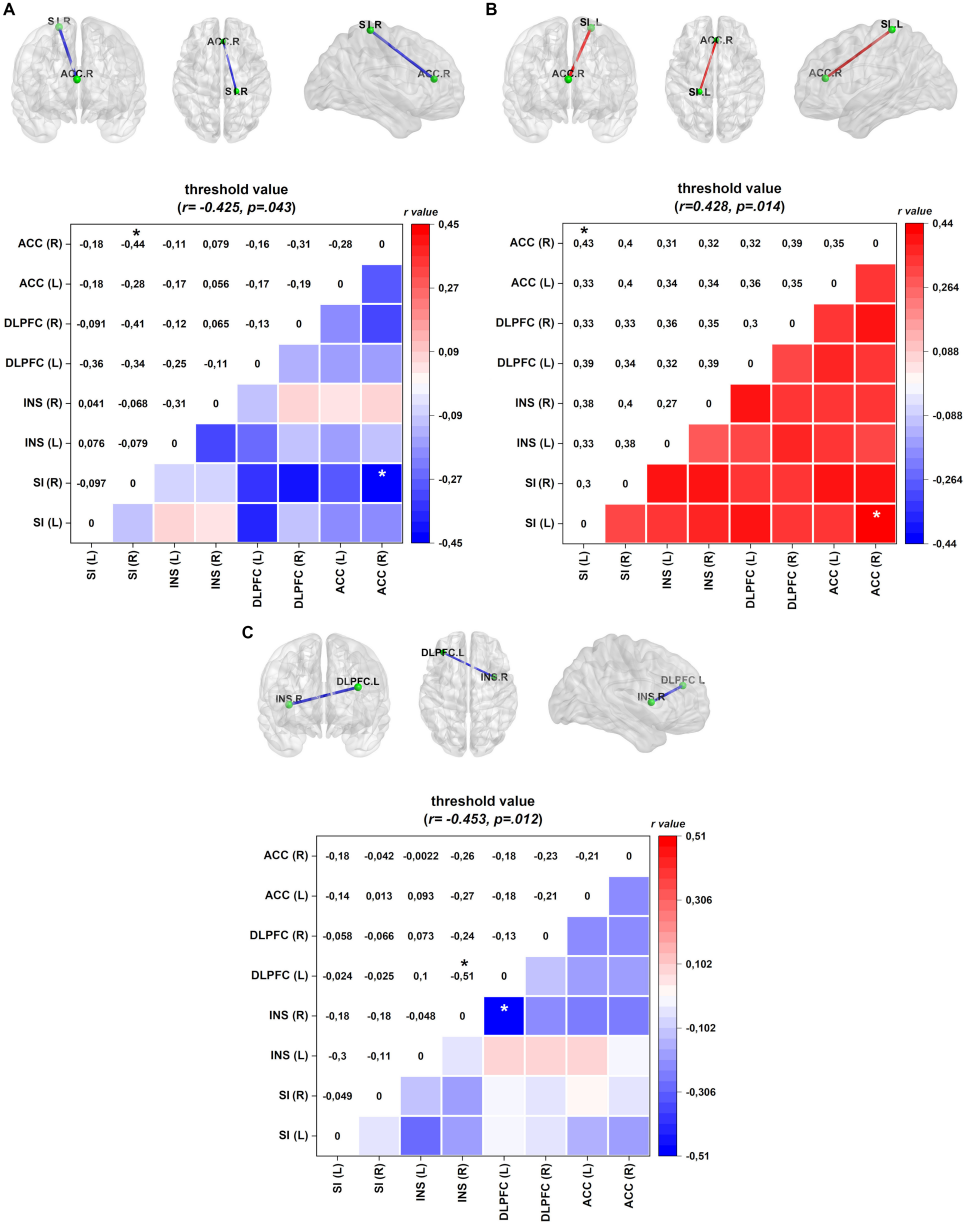
Connectivity (frontal, axial, and sagittal planes) and color maps of the linear regression analyses for FM patients between ROI connectivity and mood, pain-related symptoms, sleep quality, and biochemical measures. **(A)** In the EC condition, diminished lagged coherence connectivity in the beta-3 band between the right ACC and right INS negatively correlates with pain disability measured by FIQ. **(B)** In the EO condition, increased lagged coherence connectivity between right SI and right ACC correlates with central sensitization score in the alpha-2 band. **(C)** Serum BDNF level conversely correlates with lagged coherence connectivity between left DLPFC and right insula in the gamma band. Values given in Cohen r = correlation coefficient (small = 0.1; medium = 0.3; large = 0.5). ACC, anterior cingulate cortex; INS, insula; DLPFC, dorsolateral prefrontal cortex; SI, primary somatosensory. ^*^p < 0.05.

Linear regression analyses were conducted in FM patients to explore the relationship between ROI connectivity in distinct frequency bands and various factors, including mood, pain-related measures, sleep quality, and serum BDNF. The results are summarized in Table 5, and the corresponding patterns are shown in Figure 3.

**Table 5:** Regression analysis between ROIs and independent variables.

	Intercept	β	SD	Frequency band	*R*	*P*
**EC CONDITION**						
**Dependent variable: ROI (right-ACC ↔ right-SI)**						
Fibromyalgia Impact Questionnaire (FIQ)	7.774	−0.033	1.079	Beta-3	−0.442	0.043
**EO CONDITION**						
**Dependent variable: ROI (right-ACC ↔ left-SI)**						
Central Sensitization Inventory (CSI)	4,771	9.227	267.3	Alpha-2	0.428	0.014
**EO–EC CONDITION**						
**Dependent variable: ROI (left-DLPFC ↔ right-INS)**						
BDNF serum level	2.838	−0.046	2.587	Gamma	−0.506	0.012

Linear regression analyses for FM patients between ROI connectivity in different frequency bands for mood, pain-related, sleep quality, and biochemical measures according to EO, EC, and differences between EO and EC conditions (EO-EC; *n* = 49). β, Unstandardized coefficients; Cohen r, correlation coefficient: small = 0.1, medium = 0.3, large = 0.5. ACC, anterior cingulate cortex; INS, insula; DLPFC, dorsolateral prefrontal cortex; SI, primary somatosensory. EO, eyes-open; EC, eyes-closed. ^*^Intercept, β and SD values multiplied by (10^4^). ^**^p < 0.05.

In the EC condition, pain disability, as measured by FIQ, exhibited a negative correlation with lagged coherence connectivity in the beta-3 frequency band. FM patients with higher pain disabilities demonstrated reduced connectivity between the right ACC and the right SI. This relationship is illustrated in Figure 3A.

In the EO condition, the severity of central sensitization, as assessed by a central sensitization score, was negatively correlated with lagged coherence connectivity between the right ACC and the left SI in the alpha-2 frequency band. The details of this correlation can be found in Table 5 and Figure 3B provides a visual representation.

Furthermore, when examining the paired contrast between EO and EC conditions (EO–EC), serum BDNF levels negatively correlated with the lagged coherence connection between the left DLPFC and the right insular cortex in the gamma frequency band. This association is depicted in Figure 3C; further information can be found in Table 5.

## 4 Discussion

The main findings of this study indicate that individuals with FM showed heightened connectivity between the left DLPFC and right ACC, which are brain regions involved in attention and emotion processing during the EO condition. When comparing the conditions of EO and EC, FM patients demonstrated increased connectivity between interhemispheric insular cortices, indicating enhanced integration of sensory stimuli and the sensory-discriminative aspects of pain. Moreover, in the difference between EO and EC conditions, FM patients present increased connectivity between the left insula and right DLPFC. FM patients display distinct patterns of lagged coherence connectivity in specific brain regions, particularly in the beta-3 frequency band, during the EO condition, and in the EO–EC comparison. The beta-3 frequency band emerged as a potential marker reflecting the intricate relationship between sensory, affective, and attentional circuits in FM pain processing. In addition, the connectivity measures between SI and ACC, sensory and affective areas, were linked to pain disability and central sensitization in FM patients. Interestingly, higher levels of serum BDNF were inversely correlated with left DLPFC and the right insular cortex neural circuits involved in integrating attentional networks with pain stimuli, indicating a potential role for BDNF in modulating pain-related neural activity in FM.

These findings underscore the relevance of cerebral rhythms and provide insights into the potential of brain oscillations. They include essential details about brain oscillations and how they help different parts of the brain communicate and work together. Furthermore, they can serve as potential markers for various conditions, aiding diagnosis and developing novel therapeutic approaches. According to Alkire et al. (2008), low frequencies have a widespread distribution throughout the brain and are associated with lower arousal states such as deep sleep or anesthesia. According to the same study, higher frequencies show specific spatial interactions during high levels of alertness, such as when you are stressed or on high alert. Hauck et al. (2015) stated that the sensorimotor system and beta frequency band are closely related. These oscillations are associated with impairments in motor performance (Gilbertson et al., 2005). Furthermore, in terms of cognitive functions, beta bands play a pivotal role in visual attention, perception, emotion, and working memory (Wang, 2010). Abnormal changes in beta oscillations or beta band coherence may result in behavioral and cognitive changes (Engel and Fries, 2010). Patients with neuropathic pain exhibit an increased peak frequency in the beta band (Göschl et al., 2015; Mussigmann et al., 2022). This activity is related to sensory processing and underscores the importance of beta oscillations in chronic pain. González-Roldán et al. (2016) discovered that people with FM have a high degree of coherence in the beta-3 band (23–30 Hz) in the left hemisphere of their brains’ centroparietal areas. This result indicates a significant change in functional connectivity in the cortex resulting from sustained chronic pain. These studies show that brain frequencies, especially those in the beta band, are essential for understanding different brain states, such as sensorimotor processing, as well as how pain and other conditions affect the brain and influence people’s behavior.

In patients with FM, lagged coherence analysis revealed heightened connectivity in the beta-3 frequency band between the left DLPFC and right ACC during EO conditions (see Figure 2A). The ACC comprises the salience network and plays a role in emotional and attentional monitoring before perceiving painful stimuli. It facilitates the integration of somatosensory inputs with prefrontal areas involved in decision-making related to pain-related behaviors. The dysfunctional connectivity between pain regions and prefrontal and sensorimotor areas indicates a potential disruption in descending pain inhibition mechanisms (Flodin et al., 2014).

The results of this study suggest that DLPFC is a critical component within pain-related brain regions, including the salience network and the DPMS (Hubbard et al., 2014; Kucyi and Davis, 2015). These regions are also associated with increased connectivity within the DMN (mPFC-posterior cingulate cortex (PCC)/precuneus) or between the DMN and the PAG and periventricular gray (PVG; Kucyi et al., 2014). The dynamic communication between these systems is crucial for attentional engagement with pain (Kucyi and Davis, 2015). When considering the differences between the EO and EC conditions, the FM group exhibited an increased lagged coherence connection between the left and right insula and between the left insula and right DLPFC in the beta-3 band (see Figure 2B). Neuroimaging studies of experimentally induced acute pain have shown that the insula is a key structure for integrating sensory information with brain regions involved in cognitive, emotional, and executive functions (Bastuji et al., 2016; Adeyelu et al., 2021; Wang et al., 2022). However, the functions of the insula extend beyond pain processing because they are associated with unpleasant interoceptive and exteroceptive experiences (Craig, 2002). The right insula is believed to have a crucial role in regulating negative emotions, including pain, whereas the left insula is known to be activated during empathetic experiences across various emotions (Gu et al., 2013; Lu et al., 2016). Nonetheless, further research is necessary to explore the potential link between insula thickness, insula asymmetry, and widespread pain and their possible relationship with the severity of clinical symptoms. This would contribute to a more comprehensive understanding of these factors and their impact on the manifestation and severity of symptoms.

In the EC condition, we observed a negative correlation between the right ACC and SI in the right hemisphere, as indicated in Table 5 and Figures 3A,B. Specifically, the connectivity within the beta-3 frequency band in the right hemisphere demonstrated an inverse correlation between the impact of FM symptoms on quality of life and the connectivity between the ACC and SI. ACC, a crucial component of the limbic system, is involved in various cognitive and emotional processes. Previous studies have shown that higher levels of connectivity among cortical areas involved in pain processing are associated with decreased pain perception. ACC is particularly involved in the affective aspects of pain because the excitatory activity of its neurons contributes to the experience of negative emotions related to pain (Foland-Ross et al., 2015; Bliss et al., 2016).

During EO, we found a link between the severity of central sensitization symptoms and connectivity in the alpha-2 frequency band between the right ACC and left SI. The SI is responsible for encoding sensory aspects of pain, whereas the ACC is involved in affective processing. According to an earlier study, when patients with central sensitization are exposed to sensory stimuli, there is more neuronal activity in areas that process sensory information (de Oliveira Franco et al., 2022b). Significant structural, chemical, and functional changes have also been observed in pain-related brain areas, such as the cingulate and somatosensory cortices (Nir et al., 2008; Harte et al., 2018). How the ACC decodes and distinguishes between sensory and affective pain is unclear. According to an earlier study (Schrepf et al., 2016), in pain central sensitization, there might be a reduced antinociceptive brain response in the ACC. This finding implies that the brain’s ability to regulate and modulate pain signals could be compromised in individuals experiencing central sensitization (Caumo et al., 2017). In addition, research has demonstrated that chronic pain can enhance the connections between SI and ACC, resulting in heightened nociceptive responses and pain-aversive behaviors (Singh et al., 2020). These findings provide valuable insights into the neurobiological processes underlying FM and indicate potential avenues for utilizing techniques with the potential to remap dysfunctional neural networks. Among these techniques are transcranial electrical stimulation and transcranial magnetic stimulation. However, further studies are necessary to understand the implications of these correlations in the context of FM symptoms.

Our results show that serum BDNF levels and the lagged coherence connection in the gamma frequency band between the DLPFC and the right insula are negatively correlated (see Table 5; Figure 3C). Although the underlying mechanisms are not yet fully understood, it is plausible that gamma frequency band activity is associated with directed attention to pain in sensorimotor areas and positively correlated with increased pain intensity (Hauck et al., 2007). Additionally, individuals with chronic pain, particularly those with nociplastic pain, tend to exhibit higher levels of serum BDNF (Deitos et al., 2015; Stefani et al., 2019). Notably, serum BDNF levels have been conversely correlated with the function of DPMS (Soldatelli et al., 2021). Furthermore, elevated levels of BDNF in the spinal cord have been linked to reduced inhibitory activity of gamma-aminobutyric acid (GABA) and increased excitability of the spinothalamic tract (Spezia Adachi et al., 2015). Another study involving individuals with FM observed that a standard pain stimulus led to enhanced connectivity between motor areas and PFC (de Oliveira Franco et al., 2022a). In addition, a positive correlation was found between gamma oscillations and serum BDNF levels in response to visuotactile integration processes related to changes in human body image (Hiramoto et al., 2017). These findings shed light on the complex relationship between serum BDNF levels, gamma oscillations, and pain processing in FM. However, further research is necessary to fully comprehend the implications of these associations and their potential clinical relevance.

Our findings indicate that individuals with FM exhibit increased connectivity and activity within the beta-3 frequency band in key brain regions involved in sensory, affective, and attentional processing. Our results heightened connections and activity in the SI, ACC, and DLPFC, which are integral components of the sensorimotor, affective, and attentional circuits, respectively. The increased connectivity in these circuits in FM may reflect altered processing of pain signals and intensified integration of sensory, emotional, and attentional aspects of pain. This heightened engagement of pain-related circuits in FM could contribute to the experience of heightened stress, anxiety, and pain symptoms commonly associated with FM (Abhang et al., 2016). Although we do not have a clear explanation for these findings, they provide valuable insights into the neurobiological mechanisms underlying FM and shed light on the complex interactions between sensory, affective, and attentional processes in pain perception. It is plausible that the increased beta-3 oscillations express the current sensorimotor state involved in pain perception, regulating the affective experience of pain and promoting pain-related behavioral responses (Engel and Fries, 2010; Singh et al., 2020). Their relevance lies in improving the comprehension of these alterations in brain connectivity, which may serve as a neural marker of dysfunctional neuroplasticity and help develop therapeutic approaches for managing FM symptoms.

Several methodological aspects must be addressed in interpreting these results: *First*, this study was cross-sectional; therefore, we could not determine whether long-term chronic pain or a more severe disease was responsible for the electrophysiological changes. *Second*, the source localization has a low resolution because of the small number of EEG sensors (18 electrodes). This is sufficient for source reconstruction, but it leads to blurring of the solution and low accuracy. *Third*, the groups were not matched and had different years of schooling and ages. *Fourth*, only women were included because FM is more common in women and because men and women have different ways of dealing with pain, brain activity, and connections (Wolfe et al., 2018). *Fifth*, it is not possible to control all possible confounding factors. Antidepressants, painkillers, mood stabilizers, and antipsychotic medications are a few of these factors that affect people with FM. *Sixth,* the results should be interpreted parsimoniously because the low resolution of EEG connectivity analysis compared to other consolidated neuroimage methods to determine functional connectivity. Lastly, because this is a cross-sectional study, more longitudinal research is needed to determine the role of pain matrix connectivity as a predictor of how chronic pain will change over time.

These findings indicate that increased connectivity between different pain processing circuits, particularly in the beta-3 frequency band during rest, may serve as neural biomarkers for the chronic pain brain signature associated with neuroplasticity and the severity of FM symptoms.

## Data availability statement

The raw data supporting the conclusions of this article will be made available by the authors, without undue reservation.

## Ethics statement

The studies involving humans were approved by Research Ethics Committee at the Hospital de Clínicas de Porto Alegre. The studies were conducted in accordance with the local legislation and institutional requirements. The participants provided their written informed consent to participate in this study.

## Author contributions

RA, MZ, and WC conceived and designed the study, participated in the sequence alignment, performed the statistical analysis, and coordinated and drafted the manuscript. RA, PS, and MZ collected and registered the data. RB and RP helped in data preprocessing. IT and FF contributed to study conception and design, interpretation of results, and review of the manuscript. RA, MZ, and WC made the final review. All authors contributed to the article and approved the submitted version.
