# Supplementary material for: High-beta oscillations at EEG resting state and hyperconnectivity of pain circuitry in fibromyalgia: an exploratory cross-sectional study
Source: Front Neurosci. 2023 Nov 27;17:1233979. doi: 10.3389/fnins.2023.1233979 (PMC10712312; doi:10.3389/fnins.2023.1233979)
Supplement: Supplementary file 1 [file Data_Sheet_1.PDF]

Supplementary Table 1. Multiple Regression analysis stepwise method between significantly ROIs and independent variables including age and years of formal study in the model.

EC CONDITION					
**Dependent Variable: ROI (right-ACC ↔ right-SI)**					
Frequency Band: Beta-3					
	β	SD	t	*p*	*R*
Intercept	0.001	0.000	11.697	0.000	
Fibromyalgia Impact Questionnaire (FIQ)	-0.032*	0.000	-3.360	**0.002**	**0.440**
Age	0.201		1.554	0.127	
Years of Study	-0.152		-1.100	0.277	
**EO CONDITION**					
**Dependent Variable: ROI (right-ACC ↔ left-SI)**					
Frequency Band: Alpha-2					
	β	SD	t	*p*	*R*
Intercept	0.483	0.019	25.877	0.000	
Central Sensitization Inventory (CSI)	0.001*	0.000	2.874	**0.007**	**0.384**

Age	0.005		0.038	0.970	
Years of Study	-0.113		-0.828	0.412	
**EO-EC CONDITION**					
**Dependent Variable: ROI (left-DLPFC ↔ right-INS)**					
Frequency Band: Gamma					
	β	SD	t	*p*	*R*
Intercept	0.000	0.000	3.483	0.001	
BDNF serum level	-0.036*	0.000	-2.995	**-0.004**	**0.400**
Age	0.006		0.046	0.963	
Years of Study	0.055		0.396	0.694	

Multiple linear regression analyses for FM patients between significantly ROIs and independent variables including age and years of formal study (n = 49). β = Unstandardized coefficients; Cohen r = correlation coefficient: small=0.1, medium=0.3, large=0.5. ACC = anterior cingulate cortex; INS = insula; DLPFC = dorsolateral prefrontal cortex; S1 = primary somatosensory. EO = eyes-open; EC = eyes-closed. *β values multiplied by (10^4). **$p<0.05$.
